# Comparative effectiveness of post-discharge strategies for hospitalized smokers: Study protocol for the Helping HAND 4 randomized controlled trial

**DOI:** 10.1186/s13063-020-04257-7

**Published:** 2020-04-16

**Authors:** Nancy A. Rigotti, Kristina Schnitzer, Esa M. Davis, Susan Regan, Yuchiao Chang, Jennifer H. K. Kelley, Anna E. Notier, Karen Gilliam, Antoine Douaihy, Douglas E. Levy, Daniel E. Singer, Hilary A. Tindle

**Affiliations:** 1 Tobacco Research and Treatment Center, Massachusetts General Hospital, 100 Cambridge St., Suite 1600, Boston, MA 02114 USA; 2 Division of General Internal Medicine, Department of Medicine, Massachusetts General Hospital, Boston, MA USA; 3 Health Policy Research Center, Mongan Institute, Massachusetts General Hospital, Boston, MA USA; 4 Harvard Medical School, Boston, MA USA; 5 Department of Psychiatry, Massachusetts General Hospital, Boston, MA USA; 6 University of Pittsburgh School of Medicine, Pittsburgh, PA USA; 7 University of Pittsburgh Medical Center, Pittsburgh, PA USA; 8 Vanderbilt University Medical Center, Nashville, TN USA; 9 Geriatric Research Education and Clinical Centers (GRECC), Veterans Affairs Tennessee Valley Healthcare System, Nashville, TN USA

**Keywords:** Inpatients, Hospitalization, Smoking cessation, Nicotine dependence, Nicotine addiction, Tobacco use, Interactive voice response, Randomized controlled trial, Pharmacotherapy

## Abstract

**Background:**

Tobacco smoking remains the leading preventable cause of death in the US. A hospital admission provides smokers with a unique opportunity to stop smoking because it requires temporary tobacco abstinence while illness may enhance motivation to quit. Hospital interventions must continue post-discharge to increase tobacco abstinence long-term, but how best to accomplish this remains unclear. Building on two previous randomized controlled trials, each of which tested smoking cessation interventions that began in hospital and continued after discharge, this trial compares two interventions that provide sustained smoking cessation treatment after hospital discharge with the goal of improving long-term smoking cessation rates among hospitalized smokers.

**Methods/design:**

Helping HAND 4 is a three-site randomized controlled trial that compares the effectiveness of two active interventions for producing validated past 7-day tobacco abstinence 6 months after hospital discharge. Smokers who are admitted to three hospitals receive a standard in-hospital smoking intervention, and those who plan to quit smoking after discharge are recruited and randomly assigned to two interventions that begin at discharge, Personalized Tobacco Care Management (PTCM) or Quitline eReferral. Each lasts 3 months. At discharge, PTCM provides 8 weeks of free nicotine replacement (NRT; a participant’s choice of patch, gum, lozenge, or a combination) and then proactive smoking cessation support using an automated communication platform and live contact with a tobacco treatment specialist who is based in the health care system. In the eReferral condition, a direct referral is made from the hospital electronic health record to a community-based resource, the state’s telephone quitline. The quitline provides up to 8 weeks of free NRT and offers behavioral support via a series of phone calls from a trained coach. Outcomes are assessed at 1, 3, and 6 months after discharge. The study hypothesis is that PTCM will produce higher quit rates than eReferral.

**Discussion:**

Helping HAND 4 is a pragmatic trial that aims to evaluate interventions in real-world conditions. This project will give hospital systems critical evidence-based tools for meeting National Hospital Quality Measures for tobacco treatment and maximizing their ability to improve cessation rates and overall health for the millions of smokers hospitalized annually in the US.

**Trial registration:**

Prospectively registered prior to start of enrollment at Clinicaltrials.gov, NCT03603496 (July 27, 2018). https://register.clinicaltrials.gov/prs/app/action/SelectProtocol?sid=S00084MJ&selectaction=Edit&uid=U00002G7&ts=2&cx=ff0oxn

## Background

Cigarette smoking remains the leading preventable cause of death in the United States, and 13.7% of US adults smoked cigarettes in 2018 [1]. Each year, 8.7% or nearly 4 million adults who smoke are hospitalized. A hospital admission gives patients who smoke a unique opportunity to quit as it requires temporary abstinence from tobacco use and enhances motivation to quit, especially if the illness requiring hospitalization is smoking-related [2]. Additionally, adults who smoke tobacco may receive nicotine replacement therapy (NRT) in the hospital to reduce nicotine withdrawal symptoms, providing them an opportunity to sample an evidence-based FDA-approved cessation therapy. Patients who use NRT in the hospital are more likely to continue using it after discharge [3].

Clinical guidelines urge health care providers to address tobacco use with all hospitalized patients who use tobacco [4, 5, 50]. A robust body of evidence demonstrates that smoking cessation counseling interventions that begin in the hospital and continue for at least 1 month after discharge are effective, increasing smoking cessation rates by 37% at 6 months after discharge in a meta-analysis of 50 controlled trials, with additional benefit when NRT is added to counseling [2]. Interventions that did not continue after discharge did not produce long-term smoking cessation. This finding is consistent with a conceptual model of tobacco use as a chronic condition whose successful treatment requires sustained care [5, 6]. National Hospital Quality Measures (NHQM) adopted by the Joint Commission and Medicare aim to promote the translation of this evidence into practice. They require the routine documentation of smoking status on admission and the offer of both smoking cessation counseling and medication during the hospital stay and at discharge [4, 5].

US hospitals lack a clear blueprint for how best to implement the NHQM guidelines [2]. Sustaining smoking cessation interventions during a patient’s transition from inpatient to outpatient care is a particular challenge. Addressing this barrier is essential because over half of patients who smoke resume smoking within 3 days of hospital discharge [7], long before outpatient follow-up visits typically occur. Our research team has conducted two randomized controlled clinical trials in over 1700 patients aimed at identifying an efficient and scalable intervention that can sustain smoking cessation treatment after hospital discharge. The first trial, Helping HAND 1 [8, 9], demonstrated the effectiveness of a post-discharge Sustained Care model providing both smoking cessation counseling and medication to hospitalized patients who smoked and wanted to quit. Participants received free FDA-approved smoking cessation medication in hand at discharge and five automated phone calls over 3 months using interactive voice response (IVR) technology. Each call gave participants the option of requesting a call back from a hospital-based smoking counselor. The Sustained Care model was compared to standard discharge care, which consisted of a smoking cessation medication recommendation and advice to call the state quitline for follow-up. Sustained Care produced a 71% higher biochemically validated tobacco abstinence rates at 6 months after discharge (26% vs. 15%, *p* < 0.01), with an incremental cost-per-quit of $3217.39 [8].

The second trial, Helping HAND 2 [10, 11], aimed to improve the scalability of Sustained Care intervention by transferring the task of delivering post-discharge counseling from hospital staff to tobacco treatment specialists at a state quitline. The revised Sustained Care model increased self-reported tobacco abstinence for 3 months after discharge, compared to standard care, but produced no difference in validated tobacco abstinence rates 6 months after discharge (17% vs. 16%) [10]. In a post-hoc analysis, participants reported that the linkage from automated calls to quitline counselors was cumbersome, and fewer participants used post-discharge counseling in Helping HAND 2 than in Helping HAND 1 [11, 12]. The HH2 trial results highlighted the need to improve patients’ engagement in cessation counseling after discharge and to sustain treatment beyond the initial 3-month intervention period. A third trial, Helping HAND 3, is testing the Sustained Care model in a psychiatric hospital [13].

The current trial, Helping HAND 4, returns to the proven Sustained Care model used in Helping HAND 1, which based intervention delivery in the health care system, but it adapts the model to incorporate lessons learned from the Helping HAND 2 trial. The new intervention model, Personalized Tobacco Care Management (PTCM), aims to improve outcomes in two ways. First, it aims to increase patients’ engagement in treatment by offering patients more choices for contact than phone calls. Second, it seeks to improve care coordination with the patients’ outpatient health care team, prompting the team to continue tobacco treatment after the post-discharge transition period ends.

The Helping HAND 4 trial compares the hospital-based PTCM model to a second active treatment that connects patients to community-based cessation resources. The intervention uses an electronic referral (eReferral) made by hospital staff to the state telephone quitline for post-discharge care. Upon receiving the referral, Quitline staff will reach out to participants to offer telephone counseling and mail to them free nicotine replacement medication. Direct methods of quitline referral (e.g., eReferral) result in higher initial connection rates than less active methods [14–19] and are likely to become standard care for hospitals in the future. The PTCM and eReferral interventions vary in intensity and cost to the health care system, offering hospitals alternative strategies for meeting NHQM .

## Methods/design

### Study design

**Fig. 1:**
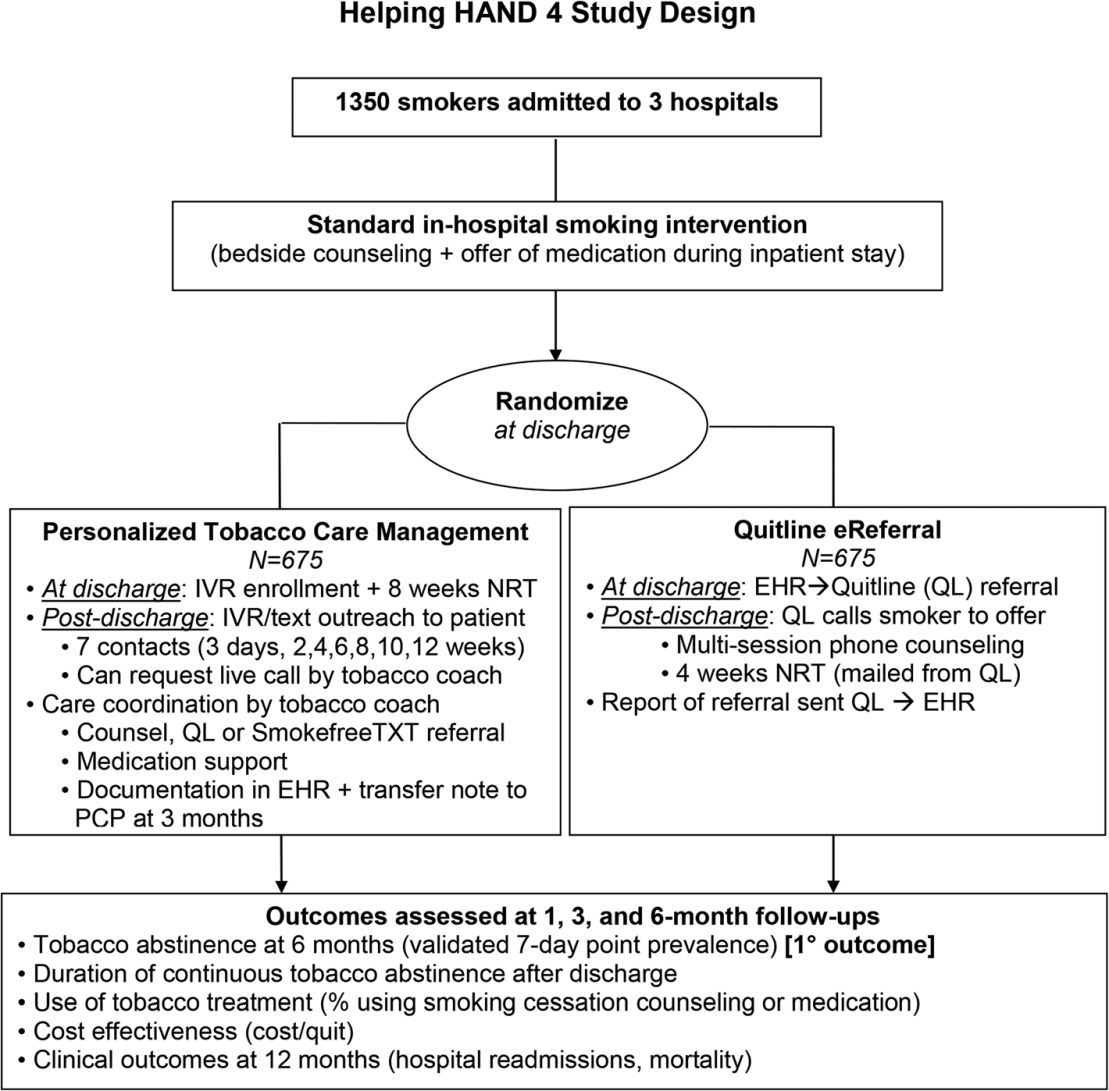
Helping HAND 4 study design. *IVR* interactive voice response, *QL* quitline, *TXT* text, *EHR* electronic health record, *NRT* nicotine replacement therapy

Helping HAND 4 is a multi-center 2-arm randomized controlled trial that compares the effectiveness and cost-effectiveness of PTCM and Quitline eReferral, two active treatments that aim to improve long-term smoking cessation rates after hospital discharge by continuing treatment that was initiated during a hospital stay (Fig. 1). Both are theoretically grounded in the Chronic Care Model [20] and are consistent with Population Health Management models of care being adopted by health care systems in response to Medicare and the Affordable Care Act [21–27].

The study hypothesis is that PTCM, compared to eReferral, will lead to more long-term tobacco cessation at 6 months post-discharge. A “usual care” or “no treatment” arm was omitted, as NHQM standards are likely to make hospitals reluctant to withhold tobacco treatment and the strength of evidence makes it unethical to do so. Further, the ubiquity of quitlines and spread of electronic health records (EHRs) makes it likely that the eReferral condition will become “standard care” in the future.

### Setting

Participants are enrolled from three large nonprofit acute care hospitals in Massachusetts, Pennsylvania, and Tennessee (Table 1). Each hospital has an established inpatient Tobacco Treatment Service (TTS) that provides a similar model of in-hospital care (Table 1). On admission, a nurse routinely documents every patient’s tobacco use status in the EHR, which generates a daily list of tobacco users to be seen by the TTS [2]. A certified tobacco treatment specialist (CTTS) attempts to visit each identified tobacco user to assess and manage nicotine withdrawal symptoms during the hospitalization as well as offer to help create a post-discharge tobacco treatment plan. The CTTS recommends a specific smoking cessation medication during hospitalization and for prescription at discharge and recommends, but does not actively make, a referral to the free state tobacco quitline. The treatment recommendation is then communicated to the hospital physician in the EHR note documenting the TTS encounter.

**Table 1:** Characteristics of the participating hospitals

Hospital *Health Care System*	MGH *Partners HealthCare*	VUMC *Vanderbilt University Medical Center*	MUH / PUH *University of Pittsburgh*
City/state	Boston, MA	Nashville, TN	Pittsburgh, PA
# Beds	1000	1004	799
# Admissions *(2015)*	50,679	46,063	39,275
Percentage male	51	46	52
Percentage white	77	71	77
Electronic health record	Epic	Epic	Cerner
Smoking counselor FTEs	3.5	2.5	3.0
NRT on formulary:	Patch, gum, lozenge, inhaler	Patch, gum, lozenge	Patch, gum, lozenge

*MGH* Massachusetts General Hospital, *VUMC* Vanderbilt University Medical Center, *MUH* Montefiore University Hospital, *PUH* Presbyterian University Hospital

### Eligibility

All adult (≥ 18 years of age) patients admitted to a study hospital who are identified in the EHR as current smokers are seen by a hospital CTTS, plan to quit or try to quit smoking after discharge, and are willing to take home a prescription for NRT are eligible for study inclusion. *Current smoking* is defined as smoking ≥ 1 cigarette/day when smoking at baseline rate in the month before admission. *Planning to quit after discharge* is operationalized by two responses to the question, “Which best describes your plan about your smoking after you leave the hospital?”, which is asked during the inpatient counseling session. Patients who select “I will stay quit” or “I will try to quit” are eligible. Those who select “I don’t know if I’m going to quit” or “I do not plan to quit” are ineligible. *Willingness to use medication* is defined by the question, “If I were to offer you nicotine patch, gum, or lozenge at no cost to you, would you agree to take it home and consider using it?” Exclusion criteria include insufficient time to complete enrollment before discharge, a patient’s inability to give informed consent or participate in counseling due to a serious cognitive or psychiatric disorder (e.g., dementia, psychosis), life expectancy < 12 months, medical instability precluding study participation, pregnancy, nursing, or planning to become pregnant within 6 months, no reliable telephone access or inability to use a telephone, lack of address to receive mail, low English proficiency, hearing/speech impairment or residence in a state whose quitline operator is not participating in the study.

### Recruitment and randomization

A multi-step process identifies eligible patients. The CTTS screens every patient counseled as a current smoker for study eligibility, informs them about the study, and requests the patient’s permission for a visit by a research team member. A research assistant (RA) visits each interested patient to describe the study, determine eligibility, obtain oral informed consent, complete the baseline assessment, and randomly assign the participant to one of two study interventions. Notification of study enrollment is sent as an EHR message to the participant’s primary care provider (PCP).

On a consent form, participants will be asked if they agree to use of their data should they choose to withdraw from the trial. Participants will also be asked for permission for the research team to share relevant data with people from the universities taking part in the research or from regulatory authorities, where relevant. This trial involves collecting biological specimens for storage at Vanderbilt University Medical Center (VUMC), with separate consent obtained for this purpose.

Randomization is stratified by study site, primary admitting diagnosis (cardiac vs. other), and cigarettes/day (≥ 10, < 10) to ensure that the treatment groups are balanced on potential confounders. Participants are randomly assigned to PTCM vs. eReferral by a computer-generated randomization scheme created by the study statistician for the corresponding stratum. The nature of the study precludes blinding of participants or research staff to the study condition. Research staff who conduct outcome assessments do not provide counseling but are aware of randomization group because follow-up assessments include program-specific evaluation questions.

### Interventions (Table 2)

#### Personalized Tobacco Care Management

The multi-component Personalized Tobacco Care Management (PTCM) model builds on the Sustained Care model tested in our previous trials [8–11, 28]. It aims to ensure that smoking cessation support and pharmacotherapy, the core components of effective tobacco dependence treatment [5], are reliably delivered to participants following hospital discharge. Additionally, it can tailor the outreach to align with participants’ preferences for mode of contact (automated phone call, text message, or email).

**Table 2:** Operationalization of treatment components of the study interventions

Treatment component	eReferral to Quitline model	Personalized Tobacco Care Management model
**Post-discharge outreach**		
Modality	Phone call from state QL	Automated phone call or text message from IVR vendor
Frequency	1 call initiated after discharge to offer QL services	7 calls made over 3 months after discharge (3 days; 2, 4, 6, 8, 10, 12 weeks)
**Free counseling**		
Services offered	5-call QL protocol (for 3 months) if participant enrolls in QL services	Up to 7 calls (over 3 months)
When offered	At initial call from QL	On demand at each of 7 IVR calls
Provider	QL-based tobacco coach	Hospital-based tobacco coach
**Free medication**		
Type	Nicotine replacement: patch ± gum ± lozenge (varies by state^a^)	Nicotine replacement: patch, gum, lozenge, inhaler or combination
Duration of treatment	4–8 weeks (varies by state^b^)	8 weeks
When provided	Mailed by QL after discharge if participant enrolls in QL services	Provided in hand at discharge
**Care coordination**	NA	Tobacco coach interfaces with PCP via EHR notes at start and end of 3-month treatment and as needed for medication prescriptions in between
**EHR integration**	Outbound: EHR referral link to QL; Inbound: varies by state^c^	Tobacco Coach notes and medication prescriptions are in EHR
**Patient choice**	NA	Patient chooses mode of contact (IVR, text, email) & treatment (phone, text)

*QL* quitline, *NJH* National Jewish Health quitline, *EHR* electronic health record, *IVR* interactive voice response, *PCP* primary care provider, *SmokefreeTXT* free NCI text messaging program

^a^ NJH serving participants in Massachusetts, Pennsylvania, and Kentucky offers patch and gum or lozenge. IQH serving Tennessee participants offers only patch

^b^ Pennsylvania increased free NRT provision to 8 weeks in July 2019. Massachusetts increased free NRT provision to 8 weeks in October 2019. Tennessee increased free NRT provision to 8 weeks for women aged 14–49 years in January 2019

^c^ VUMC site: bidirectional eReferral (results of outreach attempt sent from quitline to EHR, displayed as a note from outside provider. MGH site: outbound eReferral only (inbound result of outreach sent by fax to referring provider but not put into EHR). UPMC site: outbound only (inbound result sent electronically to EHR but does not generate EHR note)

To promote the use of cessation medication, PTCM provides study participants in hand at discharge a free 8-week supply of their choice of NRT patch, gum, or lozenge (alone or in combination). Our previous trials offered up to 3 months of any FDA-approved cessation medication (including bupropion or varenicline), with 1 month given at discharge and two refills provided on request. In the Helping HAND 2 trial, fewer than one-third of participants requested a medication refill after 1 month [28]. Providing 8 weeks of medication in hand at discharge simplifies medication delivery, may promote a longer duration of medication use and provides the 8 weeks recommended by guidelines for a full treatment course [4]. We narrowed the medication options for PTCM because NRT was the medication chosen by 95% of participants in the previous studies. Additionally, non-nicotine cessation medications require up to a week to become fully effective.

Post-discharge smoking cessation support and care coordination are conducted with an automated communication platform developed by TelASK Technologies (Ottawa, Canada) that contacts participants repeatedly in the post-discharge period and triages participants to additional counseling support. Using interactive voice response (IVR) technology, the system generates seven automated outbound phone calls at 3 days and 2, 4, 6, 8, 10, and 12 weeks after hospital discharge. Each call contains messages that promote medication adherence, support cessation efforts, and offer a return call from the hospital-based CTTS. Each automated call contact lasts 1 to 2 min. If the call fails to reach the participant, an email or text message with a link offering a return counseling call is sent, followed by another IVR call the next day. When a participant requests a return call, an attempt is made within 2 business days. At least three call attempts are made over 3 business days.

At each site, a CTTS performs the dual role of care coordinator and tobacco cessation counselor. As a coordinator, the CTTS interacts with the automated technology platform, responds to participants’ requests for return calls, and coordinates tobacco pharmacotherapy with the outpatient primary care team via EHR, fax, or email. In the counselor role, the CTTS provides brief (5–10 min) protocol-driven behavioral counseling and medication adherence support upon participant request. For example, when a participant has difficulty with the post-discharge medication, the CTTS may recommend switching the dose or contact the PCP via EHR notification or fax to request a prescription change. A detailed protocol for the phone counseling, based in motivational interviewing and cognitive-behavioral smoking cessation and relapse prevention techniques, was developed by study clinicians. The protocol also outlines medication management and adherence support, with the goal of supporting participants to complete a full course of cessation medication. Protocol modules address the following topics: choosing a medication, medication instructions and side effects, withdrawal symptoms, managing cravings, managing stress, relapse and making a new quit attempt, ambivalence, reducing negative self-talk, weight gain, rewards/self-care, and community resources.

At the end of the 12-week intervention, the study team sends a templated EHR note to the PCP to transfer care to the participant’s primary care team. The note reports the participant’s current smoking status, reviews the medication and counseling received in the past 3 months, and provides a future treatment recommendation. The recommendation includes advice to proactively contact the participant, a specific cessation medication recommendation, and advice to refer the participant to the state quitline for continued behavioral support.

#### Quitline eReferral

The control study arm actively refers a hospitalized participant to the state quitline for post-discharge care, incorporating a technologically advanced strategy that links hospital EHRs to state quitlines in a secure, HIPAA-compliant fashion. Previous studies found that referring hospitalized smokers to a quitline in a way not integrated in the EHR did not improve quit rates over usual care [29, 30]. eReferral is a less intensive, lower cost option in which smokers receive a one-time automated referral from the EHR using a secure link to the state quitline at discharge. eReferral can be used by any EHR that is compliant with Meaningful Use Stage 2, and the eReferral model has demonstrated its feasibility to engage a clinical population of hospitalized smokers at all levels of readiness to quit smoking [31, 32].

Care offered by the quitline includes behavioral support via phone counseling with a trained quitline coach and a course of free cessation medication. The number of counseling sessions offered (typically five over 3 months) and duration and type of medication (e.g., NRT for 2 to 8 weeks) are determined by each state’s quitline contract, which the state’s Department of Health negotiates with a quitline operator. In this study, two quitlines service eReferral participants. National Jewish Health (NJH) serves MA, PA, and VUMC participants residing in Kentucky. Information and Quality Healthcare (IQH) serves VUMC participants residing in Tennessee.

The study protocol planned for a bidirectional eReferral intervention at all sites; that is, a closed communication loop between the hospital and the quitline. Bidirectional eReferral follows the model of the North American Quitline Consortium formatting correction (NAQC) [32]) in which feedback reports containing information on dispositional status (i.e., reached by the quitline), smoking status, medication use, and number of counseling calls used are returned directly to the participant’s medical record. For its feasibility, interoperability and efficiency, this bidirectional model has been endorsed by NAQC as a potential national standard of care [32].

While approximately 25 state quitlines have adopted eReferral with at least one health care partner, substantial barriers to implementation of the full bidirectional model remain [33]. A major barrier is closing the eReferral loop through successful delivery of the feedback report into the participant’s EHR. This step requires the healthcare system’s IT staff to create the interface engine to automatically process and file the incoming data. Bidirectional eReferral was accomplished at VUMC but could not be implemented at the other two sites for technical reasons. At those sites, unidirectional eReferrals were electronically made from the EHR to the quitline, which sends feedback reports to study staff only.

#### Assessments

##### Baseline

The baseline survey, administered at the bedside, measures participants’ sociodemographic characteristics, current use of cigarettes, electronic cigarettes, and other tobacco products, nicotine dependence, previous tobacco cessation efforts (quit attempts and use of treatment modalities), perceived importance of quitting tobacco, confidence in ability to quit, social support for quitting, perceived health risks of smoking and benefits of quitting, anxiety symptoms, depressive symptoms, quality of life, resiliency, and general expectations about the future (i.e., dispositional optimism vs. pessimism). Alcohol and other drug use (marijuana, cocaine, opioids, stimulants, drugs by injection) are assessed for the past 30 days. The participant’s experience during hospitalization of cigarette cravings and use of cessation medications is also assessed. Participants’ past medical histories of tobacco-related diseases, discharge diagnoses, and length of stay are obtained from participants’ hospital records. Table 3 displays the details of the baseline measures.

**Table 3:** Study assessments: measures and schedule of administration

Construct	Measures	Source	Baseline	1 month	3 months	6 months
Sociodemographic factors	Age, sex, sexual orientation, education, race/ethnicity, marital status, employment, housing status, health insurance type	Patient, EHR	X			
Medical history	Coronary heart disease, chronic obstructive pulmonary disease, stroke, cancer, hypertension, diabetes, hyperlipidemia	EHR	X			
Tobacco use	*Baseline*: cigarettes per day, years smoked, other tobacco use, prior cessation attempts. *Follow-up*: duration of abstinence after discharge, cigarette and other tobacco product use in the past 7 days	Patient	X	X	X	X
Electronic cigarette use	*Baseline*: use in the past 30 days, past 7 days, frequency of use [Items from Pearson [34]], reasons for use, flavor/brand/type used. *Follow-up*: use since discharge, in past 7 days, past 30 days	Patient	X	X	X	X
Nicotine dependence	Fagerstrom Test of Nicotine Dependence (FTND) [35]	Patient	X			
Tobacco cessation treatment	*Definition*: FDA-approved cessation medications, behavioral support (telephone quit line, text-message support, in-person counseling, internet programs, mobile phone applications), other. *Baseline*: Prior use *Follow-up*: Use since discharge or since last follow-up contact	Patient	X	X	X	X
Quit attempt	*Definition*: intentional tobacco abstinence lasting > 24 h. *Baseline*: ever, past year *Follow-up*: since discharge, since last follow-up contact	Patient	X	X	X	X
Smoking cessation beliefs	Importance of quitting, confidence in ability to quit (10-point Likert scales)	Patient	X			
Perceived health risk	Risk/benefit of smoking and of quitting (5-point Likert scales)	Patient	X			
Social support for quitting smoking	10-point Likert scale	Patient	X			
Alcohol use	Alcohol Use Disorders Identification Test (AUDIT-C) [36]	Patient	X			X
Other drug use	Marijuana, cocaine, opioids, stimulants, drugs by injection (Veterans Aging Cohort Study -VACS, modified) [37]	Patient	X			X
Anxiety symptoms	Generalized Anxiety Disorder Assessment (GAD-7) [38]	Patient	X	X	X	X
Depression symptoms	Patient Health Questionnaire (PHQ-8) [39]	Patient	X	X	X	X
Quality of life	Medical Outcomes Study—Short Form (SF-1) [40]	Patient	X	X		X
Resiliency	Brief Resilience Scale (BRS) [41]	Patient	X			
Optimism, pessimism	Life Orientation Test (LOT-R) [42]	Patient	X	X	X	
Hospital experience	*Baseline*: cigarette cravings, cessation medication use in hospital *Follow-up (1 month)*: cigarette cravings, difficulty abstaining in hospital, cessation medication use and smoking in hospital	Patient	X	X		
Hospital course	Length of hospital stay, discharge diagnosis	EHR	X			
Post-discharge care coordination	PCP visit: provider’s awareness of patient’s study participation, discussion of tobacco cessation treatment	Patient		X	X	X
Health care utilization	Post-discharge ED visits, hospital re-admission (items from the National Health Interview Survey)	Patient		X	X	X
Program feedback	Services provided by study, services provided by quitline, helpfulness of services provided	Patient		X	X	X

##### Follow-up

Study participants are contacted 1, 3, and 6 months after discharge for follow-up assessments. Participants receive $20 for each completed survey. Surveys are sent by email or text if the participant consented to these contact methods. After five unsuccessful attempts, study staff call participants to administer the survey, making 24 attempts over 4 to 8 weeks. If unsuccessful, participants are mailed an abbreviated survey containing only the primary and secondary outcome measures.

Follow-up surveys assess participants’ use of cigarettes, e-cigarettes, and other tobacco products after discharge, including measures of duration of post-discharge abstinence and abstinence for the past 7 days and 30 days. Other measures include making a quit attempt (defined as intentional tobacco abstinence for > 24 h) and use of tobacco cessation treatments, including medications and behavioral support (telephone quit line, text-message support, in-person counseling, internet programs, or mobile phone applications). For each medication, duration and frequency of use and method of payment and attainment are assessed. Participants are also asked to report any hospital readmissions or emergency department visits since discharge. Baseline measures of alcohol and drug use, anxiety and depression symptoms, quality of life, and future expectations are re-assessed at follow-up. Table 3 displays the details and schedule of follow-up measures collected.

At the 1-month follow-up, participants are asked to report retrospectively how difficult it was to abstain from smoking in the hospital and whether they smoked during the inpatient stay. They are also asked if they recall receiving smoking cessation medications in hospital and/or at discharge, and to rate their satisfaction with smoking cessation assistance received in the hospital. At the 3-month follow-up (end of the active treatment period), participants are asked to rate their satisfaction with the resources provided by the study interventions.

At the 6-month survey, participants reporting tobacco abstinence in the past 7 days are asked to provide a saliva sample by mail for biochemical confirmation using an assay for cotinine, a nicotine metabolite (J2 Laboratories, Inc., Tucson, AZ, USA). Because NRT or electronic cigarette use produces a false positive cotinine result, biochemical validation for these participants requires an expired-air carbon monoxide (CO) measurement obtained at an in-person visit. Many participants who required CO verification were unable to make an in-person visit due to distance from the study sites. Consequently, the rate at which participants submitted a biochemical sample for verification was much lower for CO than for cotinine. To address this problem, we added a home-based option for CO verification in November 2019. We offer to mail participants a personalized CO device (iCO Smokerlyzer, CoVita) to complete the CO reading remotely. Participants download a free app to record CO readings that are automatically emailed to the study staff. Participants were initially provided a $50 honorarium, not contingent on test results, upon receipt of the saliva sample or completion of the CO measurement. The return rate of samples, especially CO samples, was lower than anticipated, and the honorarium was increased to $150 in January 2020.

Semi-structured qualitative telephone interviews are conducted after the final outcome assessment, using a purposive sample of participants representing both study arms and smoking outcomes. The goal is to understand in greater detail participants’ experience with the interventions and with attempting to abstain from tobacco products after discharge. Interviews are recorded, transcribed, and thematically analyzed using NVivo 12 qualitative data analysis software (QSR International Pty Ltd, version 12, 2018).

#### Outcome measures

##### Intervention effectiveness

The primary outcome measure is biochemically validated past 7-day abstinence from cigarettes and other conventional tobacco products at 6-month follow-up. As recommended by guidelines for measuring abstinence in clinical trials [43], use of e-cigarettes but no other tobacco products will be allowed in the primary outcome measure. As a sensitivity analysis, abstinence rates not allowing e-cigarette use will also be calculated [43]. Self-reported 7-day abstinence must be confirmed by saliva cotinine of ≤ 10 ng/mL or CO ≤ 9 ppm [44]. Secondary tobacco cessation outcomes include self-reported past 7-day tobacco abstinence at each follow up point (1, 3, and 6 months), repeated point-prevalence tobacco abstinence, defined as self-reported abstinence at 1, 3, and 6 months, and duration of self-reported continuous tobacco abstinence after discharge.

##### Use of tobacco treatment

Outcome measures of treatment use are the proportion of participants who report using tobacco cessation options defined as counseling and/or medication use, at 1 month and at 3 months after hospital discharge. Counseling can be provided by quitline-based tobacco coach or by a hospital-based CTTS counselor. Medication use includes use of any FDA-approved smoking cessation medication. The level of treatment engagement is measured by the number of weeks of medication use and the number of counseling contacts. Data sources include participant self-report, study records, and quitline records.

##### Cost-effectiveness

The primary cost-effectiveness analysis will assess incremental cost per quit, taking a provider organization’s perspective over the 6-month follow-up period. We will also gather data for secondary analysis taking a societal perspective. The incremental cost per quit of PTCM vs. eReferral is estimated as: (Total costs at follow-up for PTCM − Total costs at follow-up for eReferral)/(Total successful quits at follow-up for PTCM − Total successful quits at follow-up for eReferral).

The major direct costs for PTCM are development of treatment protocols and processes to connect participants to IVR/text messaging systems, CTTS effort (training, treatment delivery, supervision), and costs of the messaging services and medication/delivery. Major direct costs for eReferral are one-time information technology (IT) set-up costs and the marginal impact of eReferral on quitline budget (for secondary societal perspective analysis). We will also track post-discharge inpatient readmissions, ED visits, PCP visits, and which costs are paid by the hospital, the insurer, governments, or the participant to understand how different financing mechanisms may affect program sustainability. Indirect costs will include the value of the time the participant spends in tobacco treatment. Research costs will be excluded.

To maximize data accuracy, cost information is collected prospectively. Per unit costs for CTTS time, participant time, medication, and messaging costs are based on national average wages and prices in non-research settings. Estimating cost-effectiveness over participants’ lifetimes is beyond the scope of this study, but an exploratory analysis will assess the intervention’s potential to reduce overall resource use through 1 year after discharge*.*

##### Hospital readmissions and mortality after discharge

Exploratory analyses will assess the interventions’ effect on health and health care utilization through review of mortality and hospital readmissions for 1 year post-discharge. At each follow-up survey, we ask participants about subsequent hospital admissions, using standard items from the National Health Interview Survey. To corroborate participant reports, we review administrative data from our hospitals as well as available state-level readmission data. Among subjects lost to follow-up, mortality will be detected via proxy contacts, by reviewing hospital records, and, if necessary, the National Death Index.

##### Study fidelity and treatment integrity

Fidelity of implementation of the study protocol is important to assess in this study because it has multiple sites and the intervention includes behavioral treatment. Each site is collecting data to allow cross-site monitoring of rates of study eligibility, refusal, intervention delivery, and follow-up completion. Twice monthly calls are conducted among team members at all sites to review these data and address any discrepancies across sites.

In the PTCM arm, a counseling fidelity protocol measures post-discharge counseling within and across all three sites to ensure that counseling is delivered in accordance with the counseling modules and is documented in a standardized fashion. A random sample of 5% of CTTS counseling calls are either monitored in real time or recorded for subsequent review. Calls are coded by trained motivational interviewing adherence coders, using the Brief Intervention (BI) checklist and Motivational Interviewing Treatment Integrity (MITI) Coding Manual [45]. BI checklist items ensure that counseling is structured appropriately and contains all components of an effective brief intervention, that counseling modules discussed are relevant to participants’ needs, and that database documentation is complete. Topics assessed include statement structure and agenda setting, open motivational interviewing (MI), personalized feedback, eliciting change talk, discussion of an action plan, and closing. The MITI provides a treatment integrity measure for clinical trials incorporating MI, as well as a basis for structured, objective feedback to improve clinical practice. If a CTTS is drifting in MI technique, counseling modules, or documentation, coaching is provided and a subsequent call is monitored. Additionally, the CTTS at each site have a monthly call to discuss challenging cases and facilitate consistency in intervention delivery. Counseling in the eReferral arm is done by state quitline staff, whose performance is managed by each quitline operator’s existing quality control protocols. It is not accessible to our study staff.

#### Data analysis

##### Primary outcome

We will use an intention-to-treat analysis to preserve the integrity of randomization. Cochran-Mantel-Haenzel tests will be used to determine whether intervention effects are homogenous among three study sites. Logistic regression analysis will be used to compare the effect of study arm on the primary outcome, biochemically validated 7-day point prevalence tobacco abstinence 6 months after hospital discharge, adjusting for site. Participants who self-report smoking or whose cotinine or CO measures exceed the cut-offs will be coded as smokers. Participants who are lost to follow-up at 6 months or who report not smoking but do not provide a saliva sample or CO measurement for verification of self-report) will be coded as missing for the analysis. Following discussion with our Data Safety Monitoring Board, multiple imputation techniques will be used to estimate the missing smoking outcomes while accounting for the uncertainty from missing data.

A total sample of 1350 (675 per group) will have 84% power to detect a 6.5% absolute difference in primary outcome, verified 7-day point-prevalence abstinence at 6 months, assuming rates of 16.5% (eReferral group) and 23% (PTCM group), and a two-tailed type I error rate of 0.05. The rate ratio (1.39 = 0.23/0.165) is clinically meaningful and resembles the ratio found in meta-analysis of smoking cessation interventions for hospitalized patients [46]. We conservatively estimate the eReferral abstinence rate from the Helping HAND 2 trial (17% and 16%) [10] because participants in both conditions of that trial were referred to a quitline. For PTCM, the intervention resembles that of Helping HAND 1, where a 25% abstinence rate was observed [8], and we conservatively estimate a 23% abstinence rate.

##### Secondary outcomes

A similar analytic strategy using logistic regression with adjustment for site and other factors will be used to assess differences in self-reported point prevalence tobacco abstinence. Cross-sectional analyses will be conducted at 1, 3, and 6 months after discharge to compare outcomes between study groups. A longitudinal analysis using Generalized Estimating Equations (GEE) techniques will be used to assess the overall impact of PTCM and the time trend by including data from all follow-up times [47]. Survival analysis techniques will be used to compare self-reported days of continuous tobacco abstinence after hospital discharge. Moderator effects including discharge diagnosis (cardiac vs. other), nicotine dependence, depression symptoms, and sociodemographic factors (including sex) will be considered. In a subset of participants at the VUMC site, blood is collected to assay for nicotine and metabolites in order to calculate the nicotine metabolite ratio, a marker of hepatic nicotine metabolism [48]. The nicotine metabolite ratio will be explored as a moderator in a single-site, sub-group analysis [49].

For analysis of participant engagement in cessation treatment, regression analysis will be used to compare study arms adjusting for site and other important factors with logistic models for dichotomized outcomes (e.g., any use of medication, contact/use of medication for > 1 month), linear or Poisson models for number of contacts/weeks of use depending on the distribution of the outcome variable, and Cox proportional hazard models for duration of post-discharge medication use.

For cost-effectiveness analysis, we will use Monte Carlo simulation methods and one-way sensitivity analyses to develop confidence bounds on the incremental cost-effectiveness ratios, identify inputs with the greatest effect on cost-effectiveness, and establish the likelihood that the intervention will be feasible under a range of willingness-to-pay thresholds. If the effectiveness of PTCM is indistinguishable from that of eReferral, we will perform a cost-minimization analysis from a societal perspective.

We will compare rates of hospitalizations over 1 year between the study arms using a Poisson regression analysis. Based on the data, a Poisson mixture model such as a negative binomial distribution or a zero-inflated Poisson model will be used if there is an overdispersion issue. We will also compare time to first re-admission using a Cox proportional hazards model adjusting for site and other factors. A similar analytic plan will explore the effects on hospitalizations for cardiac and respiratory conditions. The number of factors included in the model will be subject to the number of events available.

## Discussion

Helping HAND 4 is a pragmatic trial that aims to evaluate interventions in real-world conditions. This project will give hospital systems critical evidence-based tools for meeting the new NHQMs for tobacco and maximizing their ability to improve population cessation rates and overall health for the millions of smokers hospitalized annually in the US.

## Trial status

Trial enrollment began in September 2018 at MGH and UPMC and in October 2018 at VUMC and is anticipated to conclude in spring 2020. The current study protocol is dated December 18, 2019.

## Supplementary information

**Additional file 1.**

## Data Availability

Not applicable.

## Abbreviations

BI: Brief intervention
CO: Carbon monoxide
CTTS: Certified tobacco treatment specialist
EHR: Electronic health record
GEE: Generalized Estimating Equations
IQH: Information and Quality Healthcare
IT: Information technology
IVR: Interactive voice response
MGH: Massachusetts General Hospital
MI: Motivational interviewing
MITI: Motivational interviewing treatment integrity
NAQC: North American Quitline Consortium
NHQM: National Hospital Quality Measures
NJH: National Jewish Health
NRT: Nicotine replacement therapy
PCP: Primary care provider
RA: Research assistant
TTS: Tobacco Treatment Service
UPMC: University of Pittsburgh Medical Center
US: United States
VUMC: Vanderbilt University Medical Center
