# Correction to: Comparative effectiveness of post-discharge strategies for hospitalized smokers: Study protocol for the Helping HAND 4 randomized controlled trial

**DOI:** 10.1186/s13063-020-04356-5

**Published:** 2020-05-04

**Authors:** Nancy A. Rigotti, Kristina Schnitzer, Esa M. Davis, Susan Regan, Yuchiao Chang, Jennifer H. K. Kelley, Anna E. Notier, Karen Gilliam, Antoine Douaihy, Douglas E. Levy, Daniel E. Singer, Hilary A. Tindle

**Affiliations:** 1 Tobacco Research and Treatment Center, Massachusetts General Hospital, 100 Cambridge St., Suite 1600, Boston, MA 02114 USA; 2 Division of General, Internal Medicine, Department of Medicine, Massachusetts General Hospital, Boston, MA USA; 3 Health Policy Research Center, Mongan Institute, Massachusetts General Hospital, Boston, MA USA; 4 Harvard Medical School, Boston, MA USA; 5 Department of Psychiatry, Massachusetts General Hospital, Boston, MA USA; 6 University of Pittsburgh School of Medicine, Pittsburgh, PA USA; 7 University of Pittsburgh Medical Center, Pittsburgh, PA USA; 8 Vanderbilt University Medical Center, Nashville, TN USA; 9 Geriatric Research, Education and Clinical Centers (GRECC), Veterans Affairs Tennessee Valley, Healthcare System, Nashville, TN USA

**Correction to: Trials (2020) 21: 336**

**https://doi.org/10.1186/s13063-020-04257-7**

Following publication of the original article [1], we were notified of some modifications in how the use of a specific scale for measuring treatment fidelity is described in the “Study Fidelity and Treatment Integrity” section. The paragraph should read as follows:

In the PTCM arm, a counseling fidelity protocol measures post-discharge counseling within and across all three sites to ensure that counseling is delivered in accordance with the counseling modules and is documented in a standardized fashion. A random sample of 5% of CTTS counseling calls are either monitored in real time or recorded for subsequent review. Calls are coded by trained motivational interviewing adherence coders, using the Brief Intervention (BI) checklist and **select components of the** Motivational Interviewing Treatment Integrity (MITI) Coding Manual [45]. **The** BI checklist items ensure that counseling is structured appropriately and contains all components of an effective brief intervention, that counseling modules discussed are relevant to participants’ needs, and that database documentation is complete. Topics assessed include statement structure and agenda setting, open motivational interviewing (MI), personalized feedback, eliciting change talk, discussion of an action plan, and closing. **Including select components of the** MITI provides a treatment integrity measure for clinical trials **such as this one that incorporate particular elements of** MI, as well as a basis for structured, objective feedback to improve clinical practice. If a CTTS is drifting in MI **style and skills**, counseling modules, or documentation, coaching is provided and a subsequent call is monitored. Additionally, the CTTS at each site have a monthly call to discuss challenging cases and facilitate consistency in intervention delivery. Counseling in the eReferral arm is done by state quitline staff, whose performance is managed by each quitline operator’s existing quality control protocols. It is not accessible to our study staff.

The modified parts are marked in bold.
