# Supplementary material for: Comparative effectiveness of post-discharge strategies for hospitalized smokers: Study protocol for the Helping HAND 4 randomized controlled trial
Source: Trials. 2020 Apr 16;21:336. doi: 10.1186/s13063-020-04257-7 (PMC7164139; doi:10.1186/s13063-020-04257-7)
Supplement: Supplementary file 1 — Additional file 1. [file 13063_2020_4257_MOESM1_ESM.pdf]

CHARTER

Data and Safety Monitoring Board for

NIH Grant 2R01HL111821: "Comparative Effectiveness of Post-Discharge Strategies for Hospitalized Smokers"

1. Introduction

The Charter is intended to be a living document. The DSMB may wish to review it at regular intervals to determine whether any changes in procedure are needed.

2. Responsibilities of the DSMB

Authority: Decisions of the DSMB are advisory to the trial and its investigators. However, special circumstances and documentation are required when DSMB recommendations are not followed.

Responsibilities of the DSMB: The DSMB is charged with monitoring and evaluating two aspects of the clinical trial. These include:

1. Study Progress
Screening, recruitment, and retention data will be reviewed to assure that the study can be completed in a reasonable time frame to be of significant clinical relevance.

2. Safety
Interim safety data for the trial will be reviewed in order to assure the continuing safety of subjects. As guided by Partners IRB, serious adverse events that are unexpected and related/possibly related to the research must be reported to the IRB and to NHLBI within 5 working days/ 7 calendar days of the date the investigators first become aware of them. A cumulative count of known expected and unexpected serious adverse events and a description of deaths and any unexpected serious adverse events will be reported to the DSMB during scheduled meetings.

The trial will continue to be reviewed by the DSMB until all interventions are discontinued and issues raised by the Board are concluded to the Board's satisfaction.

3. DSMB Members

The DSMB will consist of 4 members, including 2 smoking cessation investigators and 1 investigator with expertise in clinical trials. DSMB members and their expertise are listed below.

Anne Joseph, MD (Chair)
Dr. Joseph is a Wexler Professor of Medicine and general internist in the Department of Medicine. She received her medical training at the University of Michigan and received a Masters of Public Health in Epidemiology from the University of Minnesota. She co-leads the Prevention and Etiology Program at the University of Minnesota Comprehensive Cancer Center and serves as Vice Chair for Faculty Affairs and Diversity. Her primary research interests are in tobacco control. Her work has focused on smoking cessation and smoking reduction interventions for special populations of smokers that experience unique barriers to delivery of

tobacco treatment. She also works on promotion of evidence-based tobacco dependence treatment to population-based samples of smokers, using novel telephone, proactive outreach and longitudinal care interventions. Dr. Joseph is Past President of the Society for Research on Nicotine and Tobacco.

Laurel A. Beckett, PhD (Member)
Laurel A. Beckett, PhD is the Director of the UC Davis Comprehensive Cancer Center's Biostatistics Shared Resource and Chief of the Division of Biostatistics in the School of Medicine. A major theme of Dr. Beckett's research since 1987 has been the public health impact of chronic diseases of aging, especially Alzheimer's disease (AD). She led statistical efforts for the first population-based estimate of the number of people with AD in the US: 4 million. Her work led directly to increased awareness of and research funding for AD. Later papers added estimates of the annual number of new cases and the complementary impact of parkinsonism. As Director of the Alzheimer's Disease Neuroimaging Initiative (ADNI) Biostatistics Core, Dr. Beckett has also served as external advisor for three additional ADC's and for the AD prevention initiative, the CAMD biomarker initiative, the NINDS Parkinson's disease biomarker development committee, and the national Autism Biomarker Consortium for Clinical Trials. Dr. Beckett has a strong interest in Cancer Research. Her collaborations span the field from basic science to clinical trials to care-giving and community-based research. She has directed the UC Davis Comprehensive Cancer Center's Biostatistics Shared Resources since its inception in 2000. Dr. Beckett's work builds on strong mathematical training and an interest in applying discrete mathematical tools creatively to biomedical research.

John R. Hughes, MD (Member)
John R. Hughes, M.D. is Professor of Psychiatry, Psychology and Family Practice at the University of Vermont. He is Senior Associate Director of the VT Center on Tobacco Regulation Science. Dr. Hughes is board certified in Psychiatry and Addiction Psychiatry. His major focus has been clinical research on tobacco use. He is a co-founder and past president of the Society for Research on Nicotine and Tobacco, and the Association for the Treatment of Tobacco Use and Dependence. Dr. Hughes received the Ove Ferno, Alton Ochsner and the John R Hughes ATTUD Excellence in Tobacco Treatment, Training and Advocacy Award. Dr. Hughes has been Chair of the Vermont Tobacco Evaluation and Review Board which oversees VT's multi-million dollar tobacco control program. He has over 450 publications on nicotine and other drug dependencies and is one of the world's most cited tobacco scientist. Dr. Hughes has been a consultant on tobacco policy to the World Health Organization, the U.S. Food and Drug Administration, the US Office on Smoking and Health and the White House. Dr Hughes has received fees from companies who develop smoking cessation devices, medications and services, from governmental and academic institutions, and from public and private organizations that promote tobacco education or control and serves as a consultant to Swedish Match and Philip Morris for their harm-reduction products.

Harry Lando, PhD (Member)
Harry Lando, Ph.D. is internationally recognized for his work in smoking cessation and tobacco control. He has been active in this field since 1969 and has published extensively in this area with a total of over 250 scientific publications. He was a scientific editor of the 1988 Report of the US Surgeon General, The Health Consequences of Smoking: Nicotine Addiction. He was a member of the tobacco cessation clinical practice guidelines panel for the US Department of Health and Human Services and is a past member of the Center for Child Health Research Tobacco Consortium of the American Academy of Pediatrics and a past Senior Editor for Addiction. He has consulted actively with such government and voluntary agencies as the US National Heart, Lung and Blood Institute, the National Cancer Institute, the Centers for Disease

Control, the National Institute on Drug Abuse, the Agency for Healthcare Research and Quality, the American Cancer Society, the American Lung Association, the World Health Organization, and the International Union Against Tuberculosis and Lung Disease. Dr. Lando is a past president of the Society for Research on Nicotine and Tobacco and past chair of the SRNT Global Network Committee. He is a 2006 recipient of the University of Minnesota Award for Global Engagement. This award carries with it the title of "Distinguished International Professor." He was Vice President of the 14th World Conference on Tobacco OR Health held in Mumbai, India March 8-12, 2009. In 2010 he received the SRNT John Slade Award for outstanding contributions to public health and tobacco control through science-based public policy and public advocacy. He is currently chair of the Advisory Committee to the Union on the World Conferences on Tobacco OR Health (WCTOH).

4. Timing, Scheduling, and Organization of Meetings

Meetings: The DSMB will meet twice during the course of the study. The first meeting will be scheduled after one third of the target number of participants have been enrolled. The second meeting will be scheduled when half of the participants have reached the 6-month follow-up point and it will occur no later than at 48 months after start of the project. Meetings of the DSMB will be coordinated by the PI or her delegate. Three members will constitute a quorum; the members who are unable to attend will also be contacted and given an opportunity to provide input on the issues at hand after the meeting. Interim data reports will be supplied to the DSMB by the PI one week prior to each meeting. Data will be supplied in tabular or electronic forms per request of the DSMB. The investigators will create a template for the interim reports for review, edits, and approval by the DSMB chair.

Meeting Procedure: Prior to each formal meeting, it is the responsibility of the Chair of the DSMB to assure that the required data have been submitted with appropriate explanations. This material will be sent to Board members one week prior to the DSMB meeting. The formal meeting of the DSMB for the trial shall consist of three parts. The first part is an open session in which members of the research team, including the Principal Investigator or her delegate, relevant Co-Investigators and the study statisticians, are available to review data with the DSMB. Outcome results must not be discussed during this open session. Minutes from the open session will be taken by project staff. Following the open session, the DSMB may hold a closed session. The study statistician can be available to discuss the results with the DSMB during the closed session. Minutes from the closed session will be taken only by the Chair. The third phase of each meeting is optional and is a final executive session involving only voting DSMB members and may be held to allow the DSMB to discuss general conduct of the trial and all outcome results, including adverse events, to develop recommendations, and to take votes as necessary. The meeting is adjourned at the conclusion of the closed and executive sessions. Project staff will send open session minutes to the Chair within 3 working days following the meeting

5. Reports of DSMB Deliberations

Clerical support will be provided by the MGH site as requested by the Chair of the DSMB. Following each DSMB review, the Chair shall prepare a written report to be finalized within 14 working days following the formal meeting and be sent to the PI. The report will review the two main aspects of the trial for which the DSMB is responsible as noted in section 2 above (i.e., study progress and safety). In addition, following each study review, the DSMB will recommend either:

a. Continuation of the trial using the current protocol and statistical plan.
b. Continuation of the project with modifications as outlined by the Board.
c. Immediate suspension of the trial for safety reasons with a recommended plan of follow-up to minimize subject harm (requires unanimous vote).
d. Placing a clinical hold on the trial. This should include freezing further accrual. Subjects may continue on their assigned treatments until clarifications requested by the Board are resolved (requires unanimous vote).

6. Discussion of Confidential Material

No communications, either written or oral, of the deliberations or recommendations of the DSMB will be made outside of the DSMB except as provided by written policy. Study data are strictly confidential and must not be divulged to any nonmember of the Board except as indicated by policy.
